# Hypertension and the Risk of Dementia

**DOI:** 10.3389/fcvm.2020.00005

**Published:** 2020-01-31

**Authors:** Cristina Sierra

**Affiliations:** Hypertension & Vascular Risk Unit, Internal Medicine Department, Hospital Clinic of Barcelona (IDIBAPS), University of Barcelona, Barcelona, Spain

**Keywords:** essential hypertension, cognitive function, cognitive impairment, dementia, high blood pressure, antihypertensive treatment

## Abstract

Hypertension, particularly midlife high blood pressure, has been related to a higher risk of cognitive decline and dementia, including Alzheimer disease. However, these associations are complex and not fully elucidated. Cerebral small vessel disease emerges as one of the most important causes. Several observational studies have shown the potential beneficial role of antihypertensive treatment in preventing cognitive decline. However, randomized clinical trials (RCTs) have shown controversial results without proving nor disproving the association. On the other hand, in very elderly or frail people some studies have observed a relationship between low blood pressure and worse cognitive function. The optimal systolic and diastolic blood pressure values for protecting cognitive function, especially in elderly people, are not known.

## Introduction

**Figure 1:**
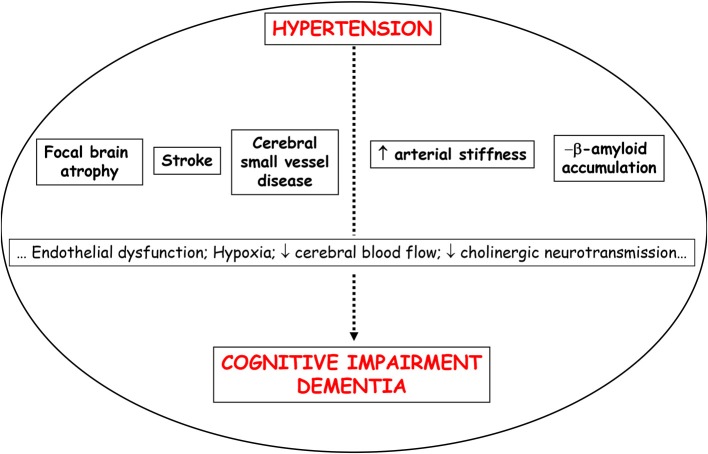
Possible mechanisms that can explain the association between hypertension and cognitive impairment/dementia.

Regardless of age, hypertension is undoubtedly the vascular risk factor (VRF) most closely related to cerebrovascular pathology (1). In fact, hypertension is the most important modifiable risk factor for stroke, lacunar infarction, cerebral white matter lesions (WML), microbleeds, cognitive impairment, and vascular dementia (1, 2) (Figure 1). Hypertension seems to predispose to early cognitive deterioration, which evolves to dementia and stroke after a time interval that may vary from a few to several years. During this time, in which the majority of hypertensives remain asymptomatic, elevated blood pressure (BP) predisposes to the development of slight alterations, founded on arteriolar narrowing or microvascular changes that lead to chronic small vessel ischemia, focal or diffuse (lacunar or WML), as well as deposits of hemosiderin in the perivascular spaces, mainly of the deep perforating arteries (microbleeds).

Cognitive impairment and dementia are increasing with the aging process of countries and provokes a huge social and economic burden (3). The main etiology of dementia is Alzheimer's disease (AD), a neurodegenerative type, and the second is vascular dementia (VD). In the last decades, some evidence support the idea that both etiologies are related to and that most patients have a mixed dementia (4). Hypertension, especially high BP during midlife, has been related with a higher risk of developing cognitive impairment and dementia (5–7). However, these associations are complex and not fully elucidated.

On the other hand, several reports have showed the association of indices of vascular aging and cognitive decline or the presence of silent cerebral small vessel disease (8, 9) (Table 1).

**Table 1:** Possible mechanisms linking elevated blood pressure to the risk of cerebrovascular disease (including cognitive impairment).

Oxidative stress
Altered endothelial function
Inflammation
Nocturnal blood pressure dipping or non-dipping
Altered renin-angiotensin system
Increased arterial stiffness
Impaired endothelial progenitor cell function
Increased brain blood barrier permeability
Less clearance of beta-amyloid
Tortuosity of white matter arterioles
Brain atrophy
Cerebral small vessel disease (White matter lesions, Lacunar infarct, Microbleeds)
Cerebral amyloid angiopathy

## Studies Linking High Blood Pressure and Cognition

### Cross-Sectional Studies

In the last decades there have been numerous studies that have shown a relationship between a decline of cognitive function and arterial hypertension across different age groups, whereas other have not shown this association. The age at which BP has been measured in studies seems to influence the risk of developing dementia. In the middle age of life (age 40–64 years) there seems to be a positive association between the elevation of BP and the presence of cognitive impairment, while in elderly population (age ≥ 65 years) this relationship is more controversial. Actually, most of cross-sectional studies performed in elderly population show that hypertension was linked to low dementia prevalence or low BP was related to high prevalence of dementia (including AD) (10).

In middle age people, the ARIC study found that hypertension in women, but not men, were related to worse performance on all cognitive tests in 13,840 individuals aged 45–69 years (11). The NHANES III showed that hypertension and diabetes mellitus, but not just hypertension, were related to worse scores in the tests of reaction time, processing speed, and working memory in 3,270 individuals aged 30–59 years (12). Sierra et al. (13) showed a relationship between the presence of silent cerebral WML and poorer performance of test of basic attention in asymptomatic, middle-aged, untreated hypertensives (mean age 54.4 years). Interestingly, in a study performed in 1,799 Chinese people, Shang et al. (14) showed that SBP, DBP, and median BP were related to cognitive deterioration (MMSE) in 40–60 years old individuals, but not in older participants (age range 40–85 years).

In elderly people, Cacciatore et al. (15) found that diastolic BP (DBP), but not systolic BP (SBP), was predictive of cognitive deterioration (MMSE) among 1,339 individuals aged 75 years and over without neurological alterations. The COGNIPRES study showed that hypertension and medication non-compliance were associated with lower MMSE scores in 1,579 individuals with an age of 71 years (16). One of the study with a larger sample, 19,386 individuals (age of assessment 65 years old), revealed that higher DBP was related to higher risk of cognitive deterioration (REGARDS study) (17).

On the contrary, the East Boston Study showed that hypertension was not related to cognitive function (attention and memory evaluation) in 3,627 individuals aged ≥ 65 years (18). The Italian Longitudinal Study on Aging, hypertension was not associated with MMSE score in 3,425 individuals aged 65–84 years (19). Interestingly, in a Swedish study performed in 500 men aged 68 years, moderate high BP (140–159 mmHg) was related to better performance of test about visuospatial and verbal skills, but severe hypertension (systolic BP ≥ 180 mmHg) was related to poorer scores on tests of memory and processing speed (20).

### Longitudinal Observational Studies

Longitudinal studies allow a better assessment for the study of the relationship in time, or causality, between hypertension and the beginning of cognitive decline.

Many studies have evaluated the relationship between high BP in midlife and the onset of dementia and AD later in life (10). Briefly, among studies with more people involved and/or longer follow-up, the Honolulu-Asia Aging Study (3,703 Japanese-American men aged ≥ 65 years and BP measured at age 45–68 years; duration: 25 years) showed a robust association between hypertension at midlife and VD and AD in late-life when the BP cut-off was 160/95 mmHg (21). Whitmer et al. (22) also showed in 8,845 people (mean age 69 years; midlife hypertension defined at mean age 42 years) that hypertension (BP ≥ 140/95 mmHg) was related to dementia. The Whitehall II Study were performed in 5,838 individuals aged 44 years at BP assessment; age at cognitive assessment was 56 years; results showed that increased SBP at baseline was related to worse performance of memory at baseline and diminished verbal fluency at follow-up, particularly in women (23). The longitudinal design of the ARIC study (13,476 individuals; follow-up 20 years) showed that baseline hypertension was related to higher deterioration in processing speed, verbal fluency, and a global composite score of cognitive functioning (24). In a Swedish study performed in 999 individuals followed 20 years (age at BP assessment 50 years), increased DBP at baseline was related to reduced cognitive function in late-life. Interestingly, that association was higher in hypertensive individuals without any antihypertensive therapy (25). Finally, the Framingham study (1,993 individuals; follow-up 28 years) revealed that among participants untreated for hypertension, SBP and DBP, as well as the proportion of visits in which hypertension was present, were inversely associated with cognitive performance. Interestingly, among subjects treated for hypertension, there was no association between cognitive functioning and longitudinally measured BP (26).

### Elderly People

In the Kungsholmen Project (27), a cohort of 1,270 individuals (aged ≥ 75 years) were followed during 6 years. In this period, 339 participants were diagnosed with dementia according to DSM-IV criteria (75% of them were AD); results revealed that individuals with higher SBP (>180 mmHg) had a relative risk (RR) of 1.5 for AD (95% CI 1.0–2.3), and 1.6 (95% CI 1.1–2.2) for dementia in general. High DBP (90 mmHg) were not related to a greater risk. Low DBP (<65 mmHg) were related to a RR of 1.7 for developing AD (95% CI 1.1–2.4), and 1.5 for dementia (95% CI 1.1–2.1) (25). Just one study (382 individuals aged 70 years; followed 15 years) showed a relationship between both increased SBP and DBP and the diagnostic of AD or dementia later (28). Results showed that individuals who developed dementia 15 years later had greater values of SBP and DBP at baseline (70 years old) compared to individuals who did not develop dementia. Results showed as well that BP diminished just before few years of the beginning of dementia and was similar to BP in non-demented subjects at this time.

The Framingham Heart Study performed in 1,423 individuals (Age at BP assessment: 66 years; Age at cognitive assessment: 71 years) showed that baseline hypertension was related to a higher memory decline 4–6 years later among men, but not among women (29). The Cardiovascular Health Study (5,888 individuals; Follow-up 7 years; Age at BP assessment ≥65 years; Age at cognitive assessment ≥72 years) showed that increased SBP was related to a deterioration in MMSE and processing speed (30).

Interestingly, the East Boston cohort study performed on slightly older individuals (3,657 individuals; Follow-up 9 years; Age at BP assessment: 74 years; Age at cognitive assessment: 83 years) showed a U-shaped association between SBP and cognitive function, and thus a SBP <130 or ≥160 mmHg was related to a greater rate of failures in the questionnaire (Short Portable Mental Status Questionnaire) (31).

On the contrary, the Chicago Health and Aging study (4,284 individuals; Follow-up 6 years; Age at BP assessment: 74 years; Age at cognitive assessment: 80 years) showed no association between BP with cognitive change (MMSE, Memory processing speed) (32).

## Hypotension and Risk of Cognitive Impairment/Dementia

As mentioned before, some studies have showed an association between low BP and the development of both AD and VD in elderly people (10). In most studies, hypotension referred to diastolic BP lower than 70 mmHg, while there were several cut-off points for systolic BP (10). Cross-sectional studies have shown controversial results while longitudinal studies showed more proofs that low BP may be a risk factor for developing dementia, including AD. In the longitudinal East Boston Study, performed in 3,789 people aged ≥ 65 years who were followed up during 3 years, a SBP ≥ 160 mmHg vs. a SBP between 130 and 139 mmHg (reference) showed an odds ratio of 0.22 (95% Confidence Interval: 0.07–0.68) for 4-year risk of AD, and a DBP <70 mmHg vs. a DBP > 90 mmHg showed an odds ratio of 1.56 (95% Confidence Interval: 0.60–4.07) (33). Nilsson et al. (34) in a study performed in older patients (599 individuals aged ≥ 80 years) followed up during 4 years, revealed that both low SBP and DBP were associated to cognitive deterioration, AD, and dementia. In this study, higher SBP was related to better cognitive scores (MMSE) (34).

The evidence of the relationship between the elevation of BP at middle age and the development of a decline in cognitive function years later is quite established, although not all the pathogenic mechanisms are known; cerebral small vessel disease is postulated as one of the main mechanisms involved. However, the chronopathology of this relationship is not sufficiently established if only elderly population is taken into account. The relationship between late-life BP and cognition may be determine by the presence and chronicity of past hypertension. In very recently published data from the ARIC study, a prospective cohort study that included 4,761 participants with 24-year follow-up and BP measurements at midlife and at late life, those with midlife and late-life hypertension (hazard ratio, 1.49) and those with midlife hypertension and late-life hypotension (hazard ratio, 1.62) had higher risk for incident dementia compared with those who remained normotensive (35).

Interestingly, the objective of a longitudinal cohort study was to identify the age at which the association between cardiovascular (CV) risk and cerebral blood flow (CBF) was strongest (36). Framingham Risk Score for CV disease, including age, sex, high-density lipoprotein cholesterol and total cholesterol values, SBP, use of antihypertensive drugs, smoking, and diabetes, were assessed during 20 years at midlife. CBF in later life was measured by pseudocontinuous arterial spin labeling magnetic resonance imaging. Results showed that CV risk at midlife were related to more reduced perfusion in the gray matter at older ages, but this relationship were not significant for CV risk in later life (36). Authors concluded that those results could advise the appropriate moment for implementing tools in order to prevent CV disease so as to be best efficient and worthwhile.

On the other hand, vascular aging *per se* promotes loss of arterial elasticity and diminished arterial compliance, which in turns can cause a reduced cerebral autoregulation. It means that the brain is more exposed to ischemic injuries when systemic BP decreases under a critical point for keeping perfusion. It is known as well that high BP affects cerebral circulation, producing adaptive vascular changes. Hypertension impacts the autoregulation of CBF by modulating the lower and upper limits of autoregulatory range toward greater BP, while hypertensives may be particularly vulnerable to episodes of hypotension (37). The dementia risk associated with the pattern of midlife hypertension and late-life hypotension may also be explained by the deleterious effect of chronic hypertension on the autoregulatory capacity of the brain. Further studies are needed to address all these issues.

## Studies Linking Antihypertensive Therapy and Cognition

### Evidence From Randomized Clinical Trials

Most of observational studies have constantly showed that reducing high blood pressure has beneficial effects in lowering the risk of cognitive impairment and dementia (3, 38).

With relation to randomized clinical trials (RCT) evidence has been conflicting. Some placebo-controlled RCT evaluated the role of antihypertensive treatment for preventing cognitive deterioration, dementia and stroke-related cognitive decline (SHEP, Syst-Eur, PROGRESS). The SHEP study showed that therapy with chlortalidone (a thiazide diuretic) in elderly individuals with isolated systolic hypertension significantly decreased the risk of stroke and CV episodes (primary objective) but not cognitive deterioration and dementia (secondary objective) (39). In the Syst-Eur trial, individuals with isolated systolic hypertension were given medical treatment, nitrendipine, and if there was no BP control, with enalapril, or hydrochlorothiazide, or both. The study found that active treatment vs. placebo decreased dementia incidence by 50% over 2 years (40). People included were kept on treatment for 2 years more in an open study. Results from the continued study strengthened the initial conclusion that prolonged antihypertensive treatment with nitrendipine diminished dementia risk by 55% (95% CI 24–73%) (41). In the PROGRESS trial (secondary prevention of stroke), the risk of dementia and cognitive impairment were assessed as a secondary objective (42). Results exhibited no significant effect of the treatment on the risk of dementia. Results just showed that therapy significantly decreased the risk of cognitive impairment and dementia with the recurrence of strokes (42).

Other studies [MOSES (43) (eprosartan vs. nitrendipine for secondary prevention of stroke); HYVET-COG (44) (perindopril/indapamide vs. placebo in very elderly people); PROFESS (45) (telmisartan vs. placebo for secondary prevention of stroke)] also showed no differences in preventing cognitive impairment or dementia with antihypertensive treatment or differences between different drugs.

Various systematic reviews and meta-analyses have controversial results about the effect of anti-hypertensive therapy on cognitive decline or dementia risk (3). It is important to note that the insufficient power of the trials to detect some effect could be due to methodological limitations: the majority of studies have used the MMSE test, that maybe is not adequate for detecting small changes in cognitive abilities (especially executive function); also most studies did not use cognition as primary endpoint; and finally time of follow-up in most studies maybe not have been long enough to notice variations in cognitive function.

### What Are the Optimal Systolic and Diastolic BP Values for Protecting Cognitive Function?

There is no clinical trial designed to analyze what is the optimal BP value for preventing cognitive decline and dementia as a primary objective. As a secondary endpoint, a sub-study of the SPRINT trial (SPRINT MIND) (46) have recently revealed that hypertensive patients randomized to achieve a SBP lower than 120 mmHg (intensive treatment; average of SBP achieved: 121.2 mmHg), compared with those randomized to achieve a SBP lower than 140 mmHg (standard treatment; average of SBP achieved: 136.2 mmHg), showed a significant lower incidence of mild cognitive decline and dementia. In this sense, the latest 2018 European Hypertension Guidelines recommend to achieve a SBP target <130 mmHg for the primary prevention of cerebrovascular damage including the prevention of cognitive decline and the incidence of dementia (47).

### Other Factors Related to High BP and Risk of Developing Cognitive Impairment

As previously mentioned, and in addition to age, various VRFs have been related to the development of a cognitive impairment (diabetes, hypercholesterolemia, smoking, obesity), with arterial hypertension due to its importance and its prevalence being the most important (1, 3, 5). It has also been observed that the greater the number of VRFs, the greater the risk (48).

There are some studies that have showed that the presence of particular aspects, or some diseases, in hypertensive population confers a greater risk of cognitive impairment. This is the case of hypertensive patients with depression (49, 50), or hypertensive population with a worse economic situation (51). In general population, many others factors such as depression, educational level, socioeconomic status (rural areas, low income, less social participation), physical inactivity or unhealthy diet have also been related to the development of cognitive impairment (3, 52–55). However, some studies are not longitudinally designed, or have short follow-up or small sample size, or the neuropsychological assessment is not complete enough. Further evidence is necessary to establish whether particular combination of some conditions confer greater risk of cognitive function impairment or if that risk is higher in hypertensive people.

On the other hand, there are some genetic factors that have been related to cognitive impairment in hypertensive population (56, 57). In the Honolulu Asia Aging Study, a longitudinal study with a follow-up of 26 years (3,065 Japanese American men), the presence of the apolipoprotein E APOEε4 allele and high systolic BP (≥160 mmHg) resulted in a greater risk of poor cognitive function compared with hypertensive patients without that allele (58). Similar results have been observed later in more studies performed in Australia (59) and American men and women from USA (60). Some polymorphisms of the ACE gene, which have been related to hypertension and cardiovascular complications in some studies (61), have been involved as well with cognitive function (57), and cerebral small vessel disease in hypertensive patients (57, 62). However, data are limited and further research are needed to explain possible interactions between genetics factors and cognitive decline in hypertensive population.

## Summary

The relationship between the elevation of BP in the middle age of life and the development of a cognitive decline years later is a fully studied and accepted fact. The role of BP on cognitive function appears to differ with age. In elderly population there are some studies that suggest that episodes of hypotension or an excessive BP reduction can cause or worsen a decline in cognitive function.

The etiopathogenic mechanisms through which an increase in BP can cause a deterioration of cognitive function are not sufficiently clarified, although among the most important is cerebral small vessel disease.

To date, RCTs performed to evaluate the effect of antihypertensive treatment on the prevention of cognitive impairment are inconclusive. It is important to note that most studies had some methodological problems. In spite of the results of the SPRINT MIND TRIAL, in elderly frail people and in very elderly population we should be cautious when treating and reducing blood pressure.

Further research is needed in order to clarify all these issues.

## Author Contributions

The author confirms being the sole contributor of this work and has approved it for publication.

### Conflict of Interest

The author declares that the research was conducted in the absence of any commercial or financial relationships that could be construed as a potential conflict of interest.
